# Epidemiology and Prognostic Factors of Herpes Simplex Virus Encephalitis in Japan: A Nationwide Database Study

**DOI:** 10.2188/jea.JE20250228

**Published:** 2026-08-05

**Authors:** Satoshi Kutsuna, Hiroyuki Ohbe, Yuya Kimura, Keito Shinmoto, Yuichiro Matsuo, Hiroki Matsui, Kiyohide Fushimi, Hideo Yasunaga

**Affiliations:** 1 Department of Infection Control, Graduate School of Medicine Faculty of Medicine, Osaka University, Osaka, Japan; 2 Department of Emergency and Critical Care Medicine, Tohoku University Hospital, Sendai, Japan; 3 Department of Clinical Epidemiology and Health Economics, School of Public Health, The University of Tokyo, Tokyo, Japan; 4 Department of Health Services Research, Graduate School of Medicine, The University of Tokyo, Tokyo, Japan; 5 Department of Health Policy and Informatics, Institute of Science Tokyo Graduate School of Medicine, Tokyo, Japan

**Keywords:** herpes simplex encephalitis, hospital mortality, Japan, incidence

## Abstract

**Background:**

Herpes simplex virus encephalitis (HSE) is a rare but life-threatening condition. While the disease has been well characterized in Western countries, large-scale epidemiological data from Japan are lacking.

**Methods:**

Using the Ministry of Health, Labour and Welfare (MHLW) Diagnosis Procedure Combination (DPC) Study Group database, we identified patients hospitalized with HSE between July 2010 and March 2023. Patients were identified using International Classification of Diseases, 10^th^ Revision codes. Nationwide incidence was estimated by multiplying the observed case counts by the annual ratio of total discharges in the national MHLW-DPC database to those in the DPC Study Group. We used generalized estimating equations to account for clustering within hospitals in multivariable models. In-hospital mortality and functional and neurological outcomes were assessed. Multivariable logistic regression identified prognostic factors.

**Results:**

A total of 6,788 patients were identified. The annual incidence of HSE ranged from 5.7 to 8.2 per 100,000 hospitalizations. Overall, in-hospital mortality was 6.0%. Of 6,378 survivors, 2,295 (36.0%) had a Barthel Index score ≤90 and 1,602 (25.1%) had impaired consciousness (Japan Coma Scale ≥1) at discharge. A composite poor outcome (death or impaired consciousness) occurred in 2,012 (29.6%). Older age (especially ≥80 years), impaired consciousness at presentation, and comorbidities such as congestive heart failure, chronic renal disease, malignancy, and underweight status were associated with in-hospital mortality and poor outcomes.

**Conclusion:**

This multicenter analysis of HSE in Japan showed that HSE was associated with substantial disability among survivors, in addition to non-negligible mortality. Our findings highlight the need for post-acute rehabilitation to reduce the burden of residual disability.

## INTRODUCTION

Herpes simplex virus (HSV) encephalitis (HSE) is the most common cause of sporadic viral encephalitis in adults worldwide.^1^^,^^2^ It is a rare but devastating disease, with an estimated annual incidence of roughly 1–3 cases per million population.^1^^,^^3^ Most adult cases are caused by HSV type 1 (HSV-1), accounting for about 90% of HSE in immunocompetent patients, whereas HSV-2 is a more frequent cause in neonates and immunocompromised hosts.^1^ HSE typically presents as an acute febrile encephalitic illness with headache, altered mental status, seizures, and focal neurological deficits (often temporal lobe dysfunction such as memory impairment or aphasia).

Despite appropriate treatment, HSE remains associated with considerable mortality and long-term neurological sequelae. Recent studies have reported acute mortality ranging from 5–20%, even with prompt acyclovir therapy,^4^^,^^5^ and a Swedish nationwide study showed a 1-year fatality rate of 14% despite universal antiviral use.^6^ Similar population-based studies from high-income countries indicate that about 10% of patients do not survive the acute phase.^7^

Several prognostic factors have been consistently linked to poor outcomes in HSE. Older age is strongly associated with higher mortality and reduced likelihood of full recovery,^8^ whereas younger patients generally fare better. Impaired consciousness at presentation—particularly coma or severe encephalopathy—is another key predictor of death or severe disability.^2^ This reflects disease severity, as shown in a multicenter study where high acute physiology scores were independently linked to poor prognosis.^9^ In addition, extensive brain involvement on magnetic resonance imaging (MRI) or elevated intracranial pressure,^2^ as well as comorbidities and immunosuppression,^8^ have been associated with worse outcomes.

While the general epidemiology and outcomes of HSE have been characterized in North America and Europe,^6^^,^^7^ there is a lack of large-scale data in the Japanese population. Most published studies from Japan have been limited to small case series or regional surveys,^10^^,^^11^ with variations in case definitions and data sources, such as prefecture-based registry data, institutional surveys, or nationwide questionnaire-based studies.^12^ This knowledge gap makes it difficult to estimate the national burden of HSE or to verify whether prognostic factors identified elsewhere hold true in the Japanese context.

Therefore, this study aimed to evaluate the incidence, in-hospital mortality, functional and neurological outcomes, and prognostic factors in Japanese patients with HSE utilizing a large-scale inpatient database covering approximately 50% of acute-care hospitalizations in Japan, the Diagnosis Procedure Combination (DPC) database.

## METHODS

### Data source

This was a retrospective cohort study using the DPC database created by the DPC Study Group, a government-funded academic collaboration.^13^ This database includes information from more than 1,000 participating acute-care hospitals, covering approximately 50% of all acute-care hospital beds in Japan.^14^ The DPC Study Group database is a large-scale inpatient administrative dataset collected from voluntarily participating acute-care hospitals in Japan, covering approximately 50% of all DPC discharges nationwide. While not inclusive of all hospitals, it includes a broad range of facilities with diverse geographic and institutional backgrounds, allowing for generalizable analyses. Based on comparisons with official reports from the Ministry of Health, Labour and Welfare (MHLW), the DPC Study Group database captured approximately 65–84% of all inpatient discharges at DPC-participating hospitals depending on the fiscal year (see eTable 1). Although not a complete census, this dataset is considered representative of acute-care hospitalizations in Japan. The database contains anonymized patient-level data, such as age, sex, diagnoses coded according to the International Classification of Diseases, 10th Revision (ICD-10), daily procedures and medications, and admission and discharge statuses. The accuracy of recorded diagnoses in the DPC database has been validated previously, demonstrating high specificity (93.2%) and moderate sensitivity (78.9%) for major diagnoses.^15^ However, validation data specific to herpes simplex encephalitis (HSE) are not available in the DPC system, and the accuracy of HSE coding in this dataset remains uncertain.

For nationwide estimation, we used publicly available aggregate statistics from the MHLW, referred to as the MHLW-DPC dataset. This dataset includes the total number of inpatient discharges from all DPC-participating hospitals in Japan but does not contain patient-level clinical information. In contrast, the DPC Study Group database is a patient-level dataset obtained from a subset of DPC hospitals that voluntarily participate in research and includes detailed clinical data, such as diagnoses, procedures, and medications.

The total number of discharges from all DPC-participating hospitals in each fiscal year was obtained from publicly available annual reports published by the MHLW. These figures were used as the denominators in nationwide incidence estimations.

### Study population

We identified patients hospitalized with HSE between July 2010 and March 2023. HSE cases were defined as those whose main diagnosis, admission precipitating diagnosis, most resource-consuming diagnosis, or second most resource-consuming diagnosis was coded with ICD-10 code B00.4 or G05.1, and whose diagnostic term corresponded to “herpesviral meningoencephalitis” or “herpesviral encephalitis” (allowing minor variations in wording). To enhance diagnostic specificity, we further required that patients received anti-herpetic therapy (eg, intravenous acyclovir) and underwent cerebrospinal fluid analysis, brain MRI, or both during their hospital stay. We defined anti-herpetic therapy as the administration of antiviral agents with activity against herpes simplex virus, including acyclovir, famciclovir, or valacyclovir. In the DPC database, each hospitalization is recorded as a separate entry, and information before or after inter-hospital transfers is not linked. Therefore, if a patient was transferred between hospitals, each stay was treated as an independent episode in our dataset.

### Patient characteristics

We collected information on demographics (age, sex), clinical status on admission (body mass index [BMI], level of consciousness), comorbidities, and hospital-related characteristics. These included ambulance transport, weekend admission (Saturday or Sunday), hospital teaching status, designation as a tertiary emergency medical center, and hospital volume (defined as the annual number of inpatients). Age was categorized into deciles (0–9, 10–19, 20–29, …, 80–89, or ≥90 years). BMI was categorized as <18.5, 18.5–24.9, 25.0–29.9, or ≥30.0 kg/m^2^, with missing values analyzed as a separate category. Consciousness level on admission was evaluated using the Japan Coma Scale (JCS) and categorized as follows: 0 (alert), 1 (delirium), 2 (somnolence), and 3 (coma), based on the initial assessment recorded at admission. Comorbidities were identified using ICD-10 codes based on the Deyo adaptation of the Charlson Comorbidity Index (see eTable 2 for code definitions). Additional variables included fiscal year and month of admission, ambulance transport, weekend admission (Saturday or Sunday), hospital teaching status, designation as a tertiary emergency medical center, and annual hospital HSE case volume. Hospital-level variables included facility characteristics available in the DPC dataset. Specifically, we used indicators for tertiary care hospitals, academic teaching function, and hospital volume (defined as the annual number of inpatients).

### Outcomes

The primary outcome was in-hospital mortality. Secondary outcomes included impaired consciousness at discharge (defined as JCS ≥1)^16^ and decreased functional status at discharge (Barthel Index ≤90).^17^ We also evaluated a composite outcome, defined as in-hospital death or impaired consciousness at discharge (JCS ≥1). Additionally, we assessed total hospitalization cost (in thousands of Japanese yen) and length of stay. Inpatient cost was defined as the total medical cost incurred during hospitalization, including bundled payments and additional fee-for-service items.

### Statistical analysis

We extrapolated from the DPC Study Group database to estimate the nationwide number of hospitalized HSE patients in Japan. For each fiscal year, we calculated the ratio of total annual discharges in the national MHLW-DPC database to those in the DPC Study Group database, then multiplied this ratio by the number of HSE cases in the Study Group database to estimate the national case count (for fiscal year 2010, which included only 9 months of data in the Study Group database, we adjusted the count to a 12-month equivalent). These estimates are limited to DPC hospitals and do not include patients treated at non-DPC hospitals; however, since DPC hospitals include nearly all large acute-care and tertiary facilities in Japan,^13^ they likely capture the vast majority of hospitalized HSE cases. The 95% confidence intervals (CIs) for nationwide estimates were calculated by applying the binomial CI for observed proportions to the national denominator.

We summarized patient characteristics using descriptive statistics and compared groups based on survival status and discharge outcomes. Categorical variables were compared using chi-square tests, and continuous variables using Student’s *t*-test or the Wilcoxon rank-sum test, as appropriate. Temporal trends in annual HSE incidence per 100,000 hospitalizations were evaluated using the Cochran–Armitage trend test. Because the DPC data are collected and released on a fiscal-year basis in Japan, all temporal analyses in this study were conducted using fiscal years, defined as April 1 through March 31 of the following year.

To identify predictors and evaluate temporal trends after adjustment, we fitted logit-link generalized estimating equation (GEE) models with an exchangeable working correlation, clustering on hospital to obtain population-averaged estimates and account for within-hospital correlation. The dependent variables were in-hospital mortality, impaired consciousness at discharge (Japan Coma Scale ≥1), poor functional status at discharge (Barthel Index ≤90), and a composite of death or impaired consciousness at discharge. Prespecified patient-level independent variables included age category, sex, BMI category, Japan Coma Scale at admission, Charlson comorbidities, and fiscal year (continuous). Hospital-level characteristics (teaching status, tertiary emergency designation, and annual inpatient volume) were entered as independent covariates to adjust for institutional characteristics. Odds ratios (ORs) and 95% confidence intervals were reported.

As a sensitivity analysis, we additionally adjusted for markers of in-hospital illness severity, including intensive care unit (ICU)/high care unit (HCU) admission, oxygen therapy, mechanical ventilation, vasopressor use, renal replacement therapy, and blood transfusion. These variables were derived from procedure codes in the DPC dataset and included in GEE models to assess whether the temporal trends in outcomes remained unchanged after adjusting for acute illness severity.

All statistical analyses were conducted using Stata/SE version 19.0 SE (StataCorp LLC, College Station, TX, USA). A two-sided *P*-value of <0.05 was considered statistically significant.

### Ethical considerations

This study was approved by the Institutional Review Board of the University of Tokyo (approval number, 3501-5; May 19, 2021). The requirement for individual informed consent was waived due to the anonymous nature of the DPC data, in accordance with national ethical guidelines in Japan.

### Data sharing statement

The datasets analyzed in this study are not publicly available due to contracts with hospitals reporting data in the database.

## RESULTS

Between July 2010 and March 2023, a total of 10,820 patients from 981 hospitals were identified in the DPC Study Group database as having been hospitalized for HSE. A flow diagram illustrating the inclusion process is provided in eFigure 1. Among them, 6,788 patients were included in the primary analysis based on the presence of anti-HSV agent administration and cerebrospinal fluid, brain imaging, or both performed during hospitalization.

**Figure 1.**
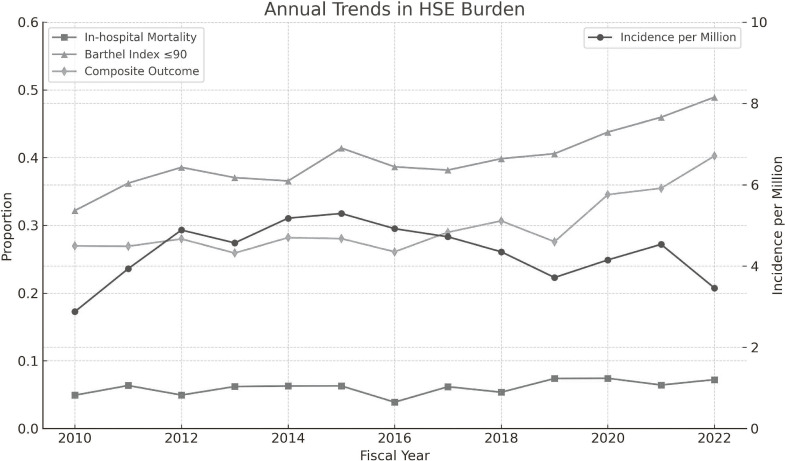
Estimated annual number of herpes simplex encephalitis (HSE) cases and estimated nationwide incidence per million population in Japan, 2010–2022.

The annual number of HSE patients ranged from 415 in fiscal year (FY)2022 to a peak of 635 in FY2015. The incidence of HSE per 100,000 hospitalizations ranged from 5.7 to 8.2 across the study period, with the highest incidence observed in fiscal year FY2012 and the lowest in FY2022 (eTable 1). A statistically significant decreasing trend was observed in the incidence of HSE per 100,000 hospitalizations from FY2012 to FY2022 (*P* for trend <0.001). The estimated number of hospitalized HSE patients in Japan, calculated based on the number of patients identified in the DPC database, ranged from approximately 620 to 794 annually throughout the study period, corresponding to 5.0 to 6.6 cases per million population per year (Figure 1).

Among the 6,788 patients, the median age was 64.0 years (interquartile range: 41.0–75.0), and 57.6% were male (Table 1). The age distribution was bimodal, with peaks in young adults (20–39 years) and older adults (60–89 years). Baseline characteristics of the study population stratified by in-hospital mortality are summarized in Table 1. Among patients who received anti-herpetic therapy, the median duration of administration was 10 days (interquartile range [IQR], 6–15 days). Of the 6,788 patients, 6,094 (89.8%) underwent cerebrospinal fluid (CSF) examination; comparisons between patients with and without CSF examination are shown in eTable 3.

**Table 1.** Patient characteristics, interventions, and outcomes of patients with HSE, overall and stratified by in-hospital survival status

Variables	Total *N* = 6,788	Survive *N* = 6,378	Death *N* = 410	*P*-value
Patients Characteristics				
Age, years, median (IQR)	64.0 (41.0–75.0)	62.5 (39.0–74.0)	77.0 (69.0–83.0)	<0.001
Age category, years, *n* (%)				
0–9	441 (6.5)	440 (6.9)	1 (0.2)	<0.001
10–19	284 (4.2)	284 (4.5)	0 (0.0)	
20–29	456 (6.7)	448 (7.0)	8 (2.0)	
30–39	496 (7.3)	490 (7.7)	6 (1.5)	
40–49	577 (8.5)	568 (8.9)	9 (2.2)	
50–59	778 (11.5)	746 (11.7)	32 (7.8)	
60–69	1,313 (19.3)	1,253 (19.6)	60 (14.6)	
70–79	1,521 (22.4)	1,377 (21.6)	144 (35.1)	
80–89	838 (12.3)	707 (11.1)	131 (32.0)	
≥90	84 (1.2)	65 (1.0)	19 (4.6)	
Male	3,913 (57.6)	3,675 (57.6)	238 (58.0)	0.86
Body mass index at admission, kg/m^2^, *n* (%)				
<18.5	976 (14.4)	891 (14.0)	85 (20.7)	0.002
18.5–24.9	3,580 (52.7)	3,370 (52.8)	210 (51.2)	
25.0–29.9	858 (12.6)	814 (12.8)	44 (10.7)	
≥30.0	196 (2.9)	190 (3.0)	6 (1.5)	
Data missing	1,178 (17.4)	1,113 (17.5)	65 (15.9)	
Japan Coma Scale at admission, *n* (%)				
Alert	1,908 (28.1)	1,818 (28.5)	90 (22.0)	<0.001
Dizziness	2,776 (40.9)	2,647 (41.5)	129 (31.5)	
Somnolence	1,089 (16.0)	1,009 (15.8)	80 (19.5)	
Coma	1,015 (15.0)	904 (14.2)	111 (27.1)	
**Comorbidities at admission, *n* (%)**				
Myocardial infarction	28 (0.4)	24 (0.4)	4 (1.0)	0.066
Congestive heart failure	172 (2.5)	141 (2.2)	31 (7.6)	<0.001
Peripheral vascular diseases	29 (0.4)	25 (0.4)	4 (1.0)	0.079
Cerebral vascular diseases	511 (7.5)	467 (7.3)	44 (10.7)	0.011
Dementia	257 (3.8)	224 (3.5)	33 (8.0)	<0.001
Chronic pulmonary diseases	188 (2.8)	165 (2.6)	23 (5.6)	<0.001
Connective tissue diseases	117 (1.7)	103 (1.6)	14 (3.4)	0.007
Peptic ulcer diseases	334 (4.9)	314 (4.9)	20 (4.9)	0.97
Liver diseases	207 (3.0)	187 (2.9)	20 (4.9)	0.026
Diabetes mellitus	1,026 (15.1)	938 (14.7)	88 (21.5)	<0.001
Chronic renal diseases	130 (1.9)	104 (1.6)	26 (6.3)	<0.001
Malignancy	279 (4.1)	224 (3.5)	55 (13.4)	<0.001
Metastasis	60 (0.9)	46 (0.7)	14 (3.4)	<0.001
Weekend admission, *n* (%)	1,509 (22.2)	1,424 (22.3)	85 (20.7)	0.45
Ambulance use, *n* (%)	3,872 (57.0)	3,597 (56.4)	275 (67.1)	<0.001
Teaching hospital, *n* (%)	6,218 (91.6)	5,864 (91.9)	354 (86.3)	<0.001
Tertiary emergency hospital, *n* (%)	3,071 (45.2)	2,906 (45.6)	165 (40.2)	0.036
Hospital case volume, mean (SD)	24.5 (24.7)	24.7 (24.8)	20.4 (22.3)	<0.001
**Organ support therapies, *n* (%)**				
ICU/HCU admission	2,220 (32.7)	2,075 (32.5)	145 (35.4)	0.24
Supplemental oxygen	3,340 (49.2)	2,983 (46.8)	357 (87.1)	<0.001
Mechanical ventilation	807 (11.9)	668 (10.5)	139 (33.9)	<0.001
Catecholamine	529 (7.8)	346 (5.4)	183 (44.6)	<0.001
Renal replacement therapy	113 (1.7)	82 (1.3)	31 (7.6)	<0.001
Blood transfusion	304 (4.5)	225 (3.5)	79 (19.3)	<0.001
**Diagnostic Tests**				
Brain MRI	6,340 (93.4)	5,952 (93.3)	388 (94.6)	0.30
CSF examination	6,094 (89.8)	5,730 (89.8)	364 (88.8)	0.49
**Outcomes**				
Japan Coma Scale at discharge, *n* (%)				
Alert	4,776 (70.4)	4,776 (74.9)	—	—
Dizziness	1,374 (20.2)	1,374 (21.5)	—	
Somnolence	149 (2.2)	149 (2.3)	—	
Coma	78 (1.1)	78 (1.2)	—	
Death	1 (0.0)	1 (0.0)	—	
Composite Outcome, *n* (%)	2,012 (29.6)	1,602 (25.1)	—	
Functional Status at discharge (Barthel Index <90), *n* (%)	2,705 (45.5)	2,295 (41.4)	—	—
Total hospitalization cost, thousand Japanese yen, median (IQR)	1,173.3 (659.3–1,975.6)	1,151.9 (650.6–1,938.5)	1,604.5 (840.1–2,837.1)	<0.001
Length of hospital stay, days, median (IQR)	25.0 (13.0–45.0)	24.0 (13.0–44.0)	28.0 (13.0–56.0)	0.004
Transferred to institutional care, *n* (%)	193 (2.8)	193 (3.0)	0 (0.0)	<0.001

BMI, body mass index; HCU, high care unit; ICU, intensive care unit; IQR, interquartile range; JCS, Japan Coma Scale.

^*^P-values were not calculated for outcomes that define the comparison groups (eg, JCS, and Barthel Index at discharge).

Overall, 410 patients (6.0%) died during hospitalization. Among the patients who died during hospitalization, the median time from admission to death was 28 days (IQR, 13.0–56.0). At discharge, 193 patients (2.8%) were transferred to institutional care settings, such as rehabilitation or nursing facilities. Compared with survivors, patients who died were older, had worse levels of consciousness at admission, and were more likely to have comorbidities such as congestive heart failure, chronic renal disease, and metastatic cancer. Among the 6,378 survivors, 2,295 (36.0%) had a Barthel Index score ≤90 at discharge, and 1,602 (25.1%) had impaired consciousness, defined as a JCS score ≥1 at discharge. A total of 2,012 (29.6%) patients met the composite outcome of death or impaired consciousness at discharge. The proportion of patients with residual functional disability (Barthel Index ≤90) increased from 32.2% (111/345) in FY2010 to 48.9% (203/415) in FY2022 (Cochran–Armitage trend test: χ^2^ = 33.2, *P* < 0.001). However, in multivariable GEE models with fiscal year as a continuous variable, this temporal trend was not statistically significant (adjusted OR per year 1.015, 95% CI 0.997–1.034; *P* = 0.105). Baseline characteristics stratified by the composite outcome and Barthel Index ≤90 at discharge are shown in eTable 4 and eTable 5, respectively.

**Figure 2.**
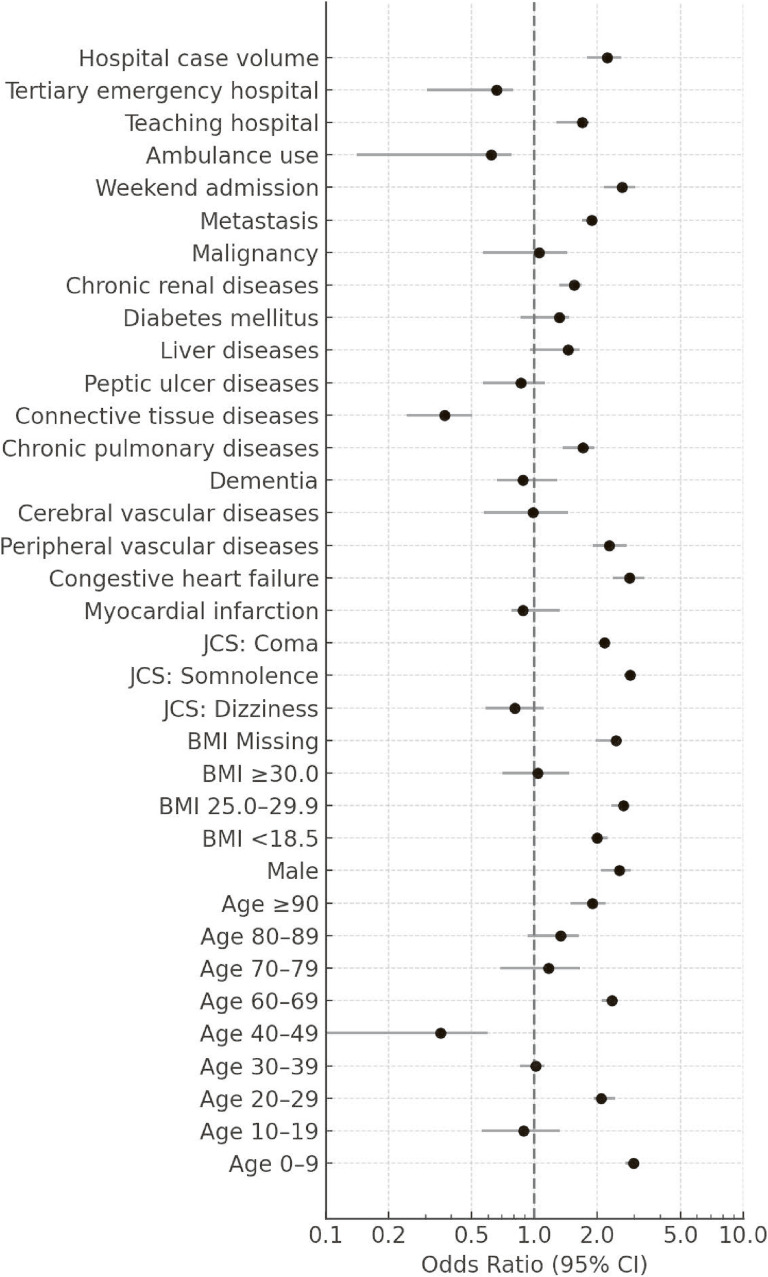
Forest plot of adjusted odds ratios for in-hospital mortality in HSE patients. Dots indicate adjusted odds ratios and lines indicate 95% confidence intervals. Note: Hospital-level variables (eg, teaching hospital status, tertiary emergency designation, hospital volume) are shown for completeness but should be interpreted differently from patient-level variables. These were included as fixed covariates in the generalized estimating equation models to account for institutional characteristics.

Multivariable logistic regression analyses using GEE showed that older age (especially ≥90 years; adjusted OR for in-hospital mortality: 4.86; 95% CI, 2.39–9.86), impaired consciousness at admission (coma vs alert: OR 1.53; 95% CI, 1.08–2.17), and comorbidities such as congestive heart failure (OR 1.96; 95% CI, 1.28–2.98), chronic renal disease (OR 2.85; 95% CI, 1.70–4.79), and metastatic cancer (OR 3.52; 95% CI, 1.83–6.80) were significantly associated with increased odds of in-hospital mortality (Table 2 and Figure 2). These factors were similarly associated with the composite outcome (eTable 6 and eFigure 2), and poor functional outcome at discharge (Barthel Index ≤90) (eTable 7 and eFigure 3), with the strongest predictors being advanced age, impaired consciousness, and multiple comorbidities.

**Table 2.** Multivariable logistic regression analysis of factors associated with in-hospital mortality among HSE patients

Variables	Odds ratio (95% CIs)	*P* value
Age 0–9	0.04 (0.01–0.38)	0.005
Age 10–19		
Age 20–29	0.47 (0.21–1.04)	0.062
Age 30–39	0.33 (0.14–0.79)	0.013
Age 40–49	0.39 (0.18–0.81)	0.012
Age 50–59 (Ref)		
Age 60–69	0.97 (0.62–1.52)	0.898
Age 70–79	2.03 (1.38–3.00)	<0.001
Age 80–89	3.40 (2.24–5.15)	<0.001
Age ≥90	4.86 (2.39–9.86)	<0.001
Male	1.22 (0.97–1.54)	0.089
BMI <18.5	1.45 (1.09–1.92)	0.011
BMI 18.5–24.9 (Ref)		
BMI 25.0–29.9	0.90 (0.63–1.29)	0.574
BMI ≥30.0	0.70 (0.30–1.64)	0.410
BMI Missing	1.31 (0.96–1.78)	0.092
JCS: Alert (Ref)		
JCS: Dizziness	0.67 (0.49–0.92)	0.013
JCS: Somnolence	0.98 (0.67–1.41)	0.899
JCS: Coma	1.53 (1.08–2.17)	0.018
Myocardial infarction	1.24 (0.40–3.90)	0.708
Congestive heart failure	1.96 (1.28–2.98)	0.002
Peripheral vascular diseases	1.11 (0.38–3.22)	0.852
Cerebral vascular diseases	0.96 (0.69–1.33)	0.790
Dementia	1.21 (0.81–1.81)	0.354
Chronic pulmonary diseases	1.63 (0.96–2.77)	0.072
Connective tissue diseases	2.00 (1.06–3.76)	0.031
Peptic ulcer diseases	1.07 (0.67–1.71)	0.781
Liver diseases	1.42 (0.86–2.35)	0.172
Diabetes mellitus	1.13 (0.88–1.45)	0.330
Chronic renal diseases	2.85 (1.70–4.79)	<0.001
Malignancy	2.73 (1.96–3.79)	<0.001
Metastasis	3.52 (1.83–6.80)	<0.001
Weekend admission	0.86 (0.67–1.11)	0.238
Ambulance use	1.17 (0.91–1.52)	0.227
Teaching hospital	0.59 (0.41–0.86)	0.006
Tertiary emergency hospital	1.04 (0.81–1.34)	0.768
Hospital case volume	0.99 (0.99–1.00)	0.221

BMI, body mass index; CI, confidence interval; JCS, Japan Coma Scale; Ref, reference category.

In sensitivity analyses additionally adjusting for markers of acute illness (ICU/HCU admission, mechanical ventilation, catecholamine use, renal replacement therapy, and transfusion), the temporal association between fiscal year and each outcome remained not statistically significant. Specifically, fiscal year was not associated with in-hospital mortality (adjusted OR 0.994; 95% CI, 0.964–1.025, *P* = 0.71), the composite outcome (adjusted OR 1.016; 95% CI, 0.998–1.035, *P* = 0.09), or Barthel Index ≤90 (adjusted OR 1.015; 95% CI, 0.997–1.034, *P* = 0.10). These results confirm that our main findings were robust regardless of illness severity adjustment.

## DISCUSSION

Although a previous Japanese study by Kamei et al proposed a prognostic scoring system based on small case series from the 1990s,^12^ large-scale contemporary data on HSE have been lacking in Japan. Using a nationwide inpatient database, this study revealed that HSE in Japan was associated with considerable in-hospital mortality and a high burden of neurological and functional sequelae.

Our analysis indicated that approximately 700 patients are hospitalized with HSE in Japan annually, corresponding to roughly 5.8 cases per million population per year. This incidence remained stable from 2010 to 2022, with unchanged mortality but gradually increasing disability. HSE is globally rare, with most Western countries reporting 2–4 cases per 1,000,000 people annually.^18^ For instance, a Swedish nationwide study (1990–2001) found an incidence of about 2.2 per million,^6^ while a more recent Danish study (2004–2014) reported 4.6 per million,^19^ suggesting improved case detection due to polymerase chain reaction-based diagnostics. Our findings in Japan (5.0–6.6 per 1,000,000/year) are at the upper end of these ranges, indicating a slightly higher HSE burden in Japan than in most Western countries. This rate is comparable to the Danish figure^19^ but higher than the older Swedish estimate,^6^ likely reflecting differences in methodology and data sources. Our nationwide administrative database included a broader hospital population than earlier registry-based or single-center studies, which may account for the higher incidence. Demographic and healthcare differences, including Japan’s aging population—where HSE disproportionately affects older adults^19^—may also contribute. Overall, the incidence of HSE in Japan appears modestly higher than in North America and Europe, underscoring the importance of vigilant diagnosis and management across regions.

Our analysis confirms that HSE significantly impacts acute survival. In our cohort, in-hospital mortality was approximately 6%, consistent with recent reports from France and other high-income countries using contemporary antiviral therapy (5–12%).^4^^,^^7^^,^^20^ This rate is lower than the historically reported 1-year fatality of 14%,^6^ likely reflecting advances in early diagnosis and prompt acyclovir use. Similarly, a recent study from Vietnam reported a 6.1% in-hospital mortality, attributed to timely antiviral administration.^21^

Among survivors in our study, many had significant disability at discharge, including low Barthel Index scores, impaired consciousness (JCS), or the need for institutional care. This aligns with international data showing that only 14–43% of patients fully recover, with most experiencing residual neurological deficits despite virologic cure.^4^^,^^9^^,^^22^ McGrath et al reported that although 70% of survivors regained independence in daily living, most still had neuropsychological impairments.^21^ A European cohort found that 35% had poor outcomes at 6 months,^9^ and a Swedish study reported high rates of rehospitalization (87%), new-onset epilepsy (24%), and neuropsychiatric complications (22%).^6^ These outcomes are consistent with our finding that many patients could not return directly home after hospitalization. A recent review emphasized that long-term issues such as memory loss, behavioral changes, and seizures are common across healthcare systems,^4^^,^^6^^,^^10^^,^^22^ highlighting the neurovirulence of HSV and its chronic impact. Many survivors require prolonged rehabilitation or nursing care, contributing to caregiver burden and healthcare costs. These impairments affect not only older adults but also working-age individuals, leading to lost productivity and independence.^6^^,^^22^ In Japan’s aging society, the combination of age-related vulnerability and high rates of disability at discharge underscores the need for early rehabilitation and coordinated discharge planning.

While in-hospital mortality remained stable, we observed a significant rise in residual disability over time, as reflected in both Barthel Index ≤90 and the composite poor outcome. This may be due to improved acute care enabling survival of more severely ill patients or demographic shifts toward older, more comorbid populations. Improved documentation may also contribute. In adjusted models, the temporal association with disability was attenuated and no longer statistically significant, suggesting case mix rather than declining care quality as the primary driver. Sensitivity analyses adjusting for illness severity (eg, ICU use, organ support) yielded consistent results. Thus, the increase in disability appears to reflect changing patient characteristics rather than treatment failure. These findings underscore the need to prioritize not only survival but also long-term function. Early diagnosis and antiviral treatment remain critical, but post-acute rehabilitation and community support are also essential to reduce the long-term burden of HSE.^23^^–^^25^

Our study identified several prognostic factors for mortality and poor outcome, largely consistent with previous international reports. Older age was a clear risk factor for in-hospital death and loss of independence, in line with prior studies showing worse HSE outcomes among elderly patients.^4^^,^^20^ Reduced physiological reserve and multiple comorbidities likely complicate recovery in this population. Severe neurological impairment on admission—reflected by deep coma or low JCS scores—was also associated with unfavorable outcomes, consistent with evidence that markers of disease severity (low Glasgow Coma Scale or JCS, extensive MRI lesions, need for intensive care) predict disability or death.^4^^,^^20^ For instance, a multicenter Turkish study found that widespread MRI lesions independently predicted poor prognosis (OR ≈37).^20^ Although MRI data were unavailable in our dataset, admission coma likely served as a surrogate for severe brain involvement. Our findings further quantify these associations in a large, real-world population: in-hospital death increased sharply with each decade of age and with worsening consciousness, paralleling trends in other HSE cohorts.^4^ This concordance with global data suggests that, despite differences in geography and healthcare systems, key prognostic factors for HSE are shared across populations. Prognostic models or risk scores developed in other countries may therefore be applicable in Japan and vice versa.

Several limitations should be noted. First, the diagnosis of HSE was based on administrative coding without microbiological confirmation. Misclassification may have occurred. Second, although we included antiviral treatment duration as a surrogate for diagnostic certainty, the accuracy of HSE diagnosis may still vary. A longer treatment course may reflect clinicians’ confidence in the diagnosis, while shorter courses might indicate empirical use that was later discontinued. Third, the lack of detailed clinical data, such as cerebrospinal fluid findings, neuroimaging results, and long-term follow-up limits the interpretation of outcomes. Fourth, while the DPC Study Group database covers a large proportion of acute-care hospitalizations, coverage varied by year (65–84%), and the sample primarily represents larger hospitals. This may affect the generalizability and precision of nationwide estimates. Finally, data on patients’ immune status or prior immunosuppressive therapy were not available, which may limit the identification of key risk factors suggested in recent literature.^26^

In conclusion, this nationwide study provides the first large-scale, real-world epidemiological analysis of HSE in Japan. Our findings demonstrate that while in-hospital mortality remains relatively low in the contemporary antiviral era, a substantial proportion of survivors experience significant functional impairments at discharge. Age, neurological severity at admission, and comorbid conditions emerged as consistent prognostic factors for poor outcomes. These results underscore the need for not only timely diagnosis and antiviral therapy but also proactive rehabilitation planning and long-term support strategies. The burden of HSE extends beyond acute care and must be addressed through integrated clinical and public health approaches.

## SUPPLEMENTARY MATERIAL

eTable 1. Annual Estimates of Herpes Simplex Encephalitis Hospitalizations and Incidence in Japan, 2010–2022

eTable 2. ICD-10 Codes Used to Define Comorbidities Based on the Charlson Comorbidity Index

eTable 3. Baseline characteristics of patients stratified by the composite outcome (in-hospital death or impaired consciousness at discharge)

eTable 4. Baseline characteristics of patients stratified by functional outcome (Barthel Index ≤90 at discharge)

eTable 5. Comparison of Baseline Characteristics, Treatments, and Outcomes Between HSE Patients With and Without Cerebrospinal Fluid (CSF) Examination

eTable 6. Multivariable logistic regression analysis of predictors for the composite outcome (death or impaired consciousness at discharge)

eTable 7. Multivariable logistic regression analysis of predictors for poor functional outcome (Barthel Index ≤90 at discharge)

eFigure 1. Flow diagram of patient selection for the study cohort. Patients hospitalized with viral encephalitis or bacterial meningitis were screened using ICD-10 codes and diagnostic text. We excluded patients whose diagnostic text was not consistent with herpes simplex encephalitis (HSE), those who did not receive anti-herpetic treatment, and those who underwent neither cerebrospinal fluid (CSF) examination nor brain magnetic resonance imaging (MRI). The final study cohort included 6,788 patients.

eFigure 2. Forest plot of adjusted odds ratios for the composite outcome in patients with herpes simplex encephalitis. Dots indicate adjusted odds ratios and horizontal lines indicate 95% confidence intervals. The composite outcome was defined as in-hospital death or impaired consciousness at discharge (Japan Coma Scale [JCS] ≥1). Estimates were obtained from generalized estimating equation logistic regression models accounting for within-hospital clustering. Hospital-level variables were included as fixed covariates to adjust for institutional characteristics.

eFigure 3. Forest plot of adjusted odds ratios for poor functional outcome at discharge in patients with herpes simplex encephalitis. Dots indicate adjusted odds ratios and horizontal lines indicate 95% confidence intervals. Poor functional outcome was defined as a Barthel Index ≤90 at discharge. Estimates were obtained from generalized estimating equation logistic regression models accounting for within-hospital clustering. Hospital-level variables were included as fixed covariates to adjust for institutional characteristics.
