# Supplementary material for: Epidemiology and Prognostic Factors of Herpes Simplex Virus Encephalitis in Japan: A Nationwide Database Study
Source: J Epidemiol. 2026 Aug 5;36(8):257–64. doi: 10.2188/jea.JE20250228 (PMC13349789; doi:10.2188/jea.JE20250228)
Supplement: Supplementary file 1 [file je-36-257-s001.pdf]

eTable 1. Annual Estimates of Herpes Simplex Encephalitis Hospitalizations and Incidence in Japan, 2010–2022
CI, confidence interval; DPC, Diagnosis Procedure Combination.

Year	The number of Herpes Encephalitis Patients in DPC database	hospitalization in DPC database	Number of Herpes Encephalitis Patients per 100000 Hospitalization in DPC database	Number of patients discharged from hospitals with DPC overall	Estimated Number of Hospitalized Patients with Herpes Encephalitis in Japan	95% CI	Estimated number of hospitalizations for herpes encephalitis per million population in Japan	95% CI
2010	460	6,056,096	7.60	8,293,897	630.0	570.8–689.2	5.2	4.8–5.7
2011	472	6,779,693	6.96	8,580,272	597.4	426.6–522.6	5.0	3.6–4.4
2012	586	7,149,269	8.20	8,981,415	736.2	520.3–625.4	6.1	4.3–5.2
2013	548	7,110,671	7.71	9,205,620	709.5	484.3–587.6	5.9	4.0–4.9
2014	621	8,070,942	7.69	9,606,676	739.2	502.0–602.5	6.2	4.2–5.0
2015	635	8,216,441	7.73	9,912,207	766.1	538.8–643.5	6.4	4.5–5.4
2016	590	8,542,884	6.91	10,114,812	698.6	482.4–580.8	5.8	4.0–4.8
2017	566	8,266,247	6.85	10,215,878	699.5	503.6–606.2	5.8	4.2–5.1
2018	522	8,233,259	6.34	10,495,933	665.5	444.2–542.5	5.5	3.7–4.5
2019	446	7765499	5.74	10800690	620.3	401.6–499.7	5.2	3.3–4.2
2020	498	7395465	6.73	10416146	701.4	470.7–577.2	5.8	3.9–4.8
2021	544	7,163,057	7.59	10,453,023	793.9	534.8–650.1	6.6	4.5–5.4
2022	415	7,128,498	5.82	11,038,857	642.6	395.9–499.1	5.4	3.3–4.2

eTable 2. ICD-10 Codes Used to Define Comorbidities Based on the Charlson Comorbidity Index

AIDS, acquired immune deficiency syndrome; HIV, human immunodeficiency virus; ICD-10, International Classification of Diseases, 10th Revision.

**Comorbidity**	**ICD-10 Codes**
Myocardial infarction	I21, I22, I252
Congestive heart failure	I43, I50, I099, I110, I130, I132, I255, I420, I425, I426, I427, I428, I429, P290
Peripheral vascular disease	I70, I71, I731, I738, I739, I771, I790, I792, K551, K558, K559, Z958, Z959
Cerebrovascular disease	I6, G45, G46, H340
Dementia	F00, F01, F02, F03, G30, F051, G311
Chronic pulmonary disease	J4, J60, J61, J62, J63, J64, J65, J66, J67, I278, I279, J684, J701, J703
Rheumatic disease	M05, M06, M32, M33, M34, M315, M351, M353, M360
Peptic ulcer disease	K25, K26, K27, K28
Mild liver disease	B18, K73, K74, K700, K701, K702, K703, K709, K713, K714, K715, K717, K760, K762, K763, K764, K768, K769, Z944
Diabetes without complication	E100, E101, E106, E108, E109, E110, E111, E116, E118, E119, E120, E121, E126, E128, E129, E130, E131, E136, E138, E139, E140, E141, E146, E148, E149
Diabetes with complication	E102, E103, E104, E105, E107, E112, E113, E114, E115, E117, E122, E123, E124, E125, E127, E132, E133, E134, E135, E137, E142, E143, E144, E145, E147
Hemiplegia or paraplegia	G81, G82, G041, G114, G801, G802, G830, G831, G832, G833, G834, G839
Renal disease	N18, N19, I120, I131, N032, N033, N034, N035, N036, N037, N052, N053, N054, N055, N056, N057, N250, Z490, Z491, Z492, Z940, Z992
Malignancy	C0, C1, C2, C3, C5, C6, C9, C40, C41, C43, C45, C46, C47, C48, C49, C70, C71, C72, C73, C74, C75, C76, C81, C82, C83, C84, C85, C88
Severe liver disease	I85, I864, I982, K704, K711, K721, K729, K765, K766, K767
Metastatic solid tumor	C77, C78, C79, C80
HIV/AIDS	B20, B21, B22, B24

eTable 3. Comparison of Baseline Characteristics, Treatments, and Outcomes Between HSE Patients With and Without Cerebrospinal Fluid (CSF) Examination

**Variables**		Total	Without CSF	With CSF	*P*-value
		N=6,788	N=694	N=6,094	
Patients Characteristics					
Age, years, median (IQR)		64.0 (41.0–75.0)	66.5 (46.0–78.0)	63.0 (40.0–75.0)	0.001
Age category, years, n (%)	0–9	441 (6.5)	52 (7.5)	389 (6.4)	<0.001
	10–19	284 (4.2)	28 (4.0)	256 (4.2)	
	20–29	456 (6.7)	36 (5.2)	420 (6.9)	
	30–39	496 (7.3)	31 (4.5)	465 (7.6)	
	40–49	577 (8.5)	58 (8.4)	519 (8.5)	
	50–59	778 (11.5)	81 (11.7)	697 (11.4)	
	60–69	1,313 (19.3)	113 (16.3)	1,200 (19.7)	
	70–79	1,521 (22.4)	172 (24.8)	1,349 (22.1)	
	80–89	838 (12.3)	104 (15.0)	734 (12.0)	
	>90	84 (1.2)	19 (2.7)	65 (1.1)	
Male		3,913 (57.6)	364 (52.4)	3,549 (58.2)	0.003
Body mass index at admission, kg/m^2, n (%)	<18.5	976 (14.4)	115 (16.6)	861 (14.1)	0.45
	18.5–24.9	3,580 (52.7)	348 (50.1)	3,232 (53.0)	
	25.0–29.9	858 (12.6)	87 (12.5)	771 (12.7)	
	≥30.0	196 (2.9)	20 (2.9)	176 (2.9)	
	Data missing	1,178 (17.4)	124 (17.9)	1,054 (17.3)	
Japan Coma Scale at admission, n (%)	Alert	1,908 (28.1)	227 (32.7)	1,681 (27.6)	<0.001
	Dizziness	2,776 (40.9)	260 (37.5)	2,516 (41.3)	
	Somnolence	1,089 (16.0)	103 (14.8)	986 (16.2)	
	Coma	1,015 (15.0)	104 (15.0)	911 (14.9)	
**Comorbidities at admission, n (%)**					
Myocardial infarction		28 (0.4)	6 (0.9)	22 (0.4)	0.050
Congestive heart failure		172 (2.5)	22 (3.2)	150 (2.5)	0.26
Peripheral vascular diseases		29 (0.4)	8 (1.2)	21 (0.3)	0.002
Cerebral vascular diseases		511 (7.5)	64 (9.2)	447 (7.3)	0.074
Dementia		257 (3.8)	41 (5.9)	216 (3.5)	0.002
Chronic pulmonary diseases		188 (2.8)	18 (2.6)	170 (2.8)	0.77
Connective tissue diseases		117 (1.7)	13 (1.9)	104 (1.7)	0.75
Peptic ulcer diseases		334 (4.9)	31 (4.5)	303 (5.0)	0.56

Liver diseases		207 (3.0)	30 (4.3)	177 (2.9)	0.040
Diabetes mellitus		1,026 (15.1)	83 (12.0)	943 (15.5)	0.014
Chronic renal diseases		130 (1.9)	22 (3.2)	108 (1.8)	0.011
Malignancy		279 (4.1)	41 (5.9)	238 (3.9)	0.012
Metastasis		60 (0.9)	4 (0.6)	56 (0.9)	0.36
Weekend admission, n (%)		1,509 (22.2)	177 (25.5)	1,332 (21.9)	0.029
Ambulance use, n (%)		3,872 (57.0)	389 (56.1)	3,483 (57.2)	0.58
Teaching hospital, n (%)		6,218 (91.6)	601 (86.6)	5,617 (92.2)	<0.001
Tertiary emergency hospital, n (%)		3,071 (45.2)	258 (37.2)	2,813 (46.2)	<0.001
Hospital case volume, mean (SD)		24.5 (24.7)	19.6 (22.2)	25.0 (24.9)	<0.001
**Organ support therapies, n (%)**					
ICU/HCU admission		2,220 (32.7)	160 (23.1)	2,060 (33.8)	<0.001
Supplemental oxygen		3,340 (49.2)	324 (46.7)	3,016 (49.5)	0.16
Mechanical ventilation		807 (11.9)	58 (8.4)	749 (12.3)	0.002
Catecholamine		529 (7.8)	44 (6.3)	485 (8.0)	0.13
Renal replacement therapy		113 (1.7)	15 (2.2)	98 (1.6)	0.28
Blood transfusion		304 (4.5)	36 (5.2)	268 (4.4)	0.34
**Diagnostic Tests**					
brain MRI		6,340 (93.4)	694 (100.0)	5,646 (92.6)	<0.001
CSF examination		6,094 (89.8)	0 (0.0)	6,094 (100.0)	
**Outcomes**					
In-hospital mortality (%)		410 (6.0)	46 (6.6)	364 (6.0)	0.49
Japan Coma Scale at discharge, n (%)	Alert	4,776 (70.4)	495 (71.3)	4,281 (70.2)	0.53
	Dizziness	1,374 (20.2)	126 (18.2)	1,248 (20.5)	
	Somnolence	149 (2.2)	20 (2.9)	129 (2.1)	
	Coma	78 (1.1)	7 (1.0)	71 (1.2)	
	Death	1 (0.0)	0 (0.0)	1 (0.0)	
Composite outcome, n (%)		2,012 (29.6)	199 (28.7)	1,813 (29.8)	0.56
Functional Status at discharge (Barthel Index <90), n (%)		2,705 (45.5)	289 (49.5)	2,416 (45.0)	0.039
Total hospitalization cost, thousand Japanese yen, median (IQR)		1173.3 (659.3–1975.6)	1011.5 (520.5–1790.4)	1182.5 (674.8–1990.4)	<0.001
Length of hospital stay, days, median (IQR)		25.0 (13.0–45.0)	22.0 (11.0–44.0)	25.0 (13.0–45.0)	0.005
Transferred to institutional care, n(%)		193 (2.8)	26 (3.7)	167 (2.7)	0.13

HCU, high care unit; ICU, intensive care unit; IQR, interquartile range; MRI, magnetic resonance imaging.

**P*-values were not calculated for outcomes that define the comparison groups (eg, in-hospital mortality, Japan Coma Scale, and Barthel Index at discharge).

eTable 4. Baseline characteristics of patients stratified by the composite outcome (in-hospital death or impaired consciousness at discharge)

**Variables**		Total	Without Composite Outcome	Composite Outcome	*P*-value
		N=6,788	N=4,776	N=2,012	
Patients Characteristics					
Age, years, median (IQR)		64.0 (41.0–75.0)	58.0 (33.0–72.0)	73.0 (62.0–80.0)	<0.001
Age category, years, n (%)	0–9	441 (6.5)	418 (8.8)	23 (1.1)	<0.001
	10–19	284 (4.2)	262 (5.5)	22 (1.1)	
	20–29	456 (6.7)	409 (8.6)	47 (2.3)	
	30–39	496 (7.3)	422 (8.8)	74 (3.7)	
	40–49	577 (8.5)	482 (10.1)	95 (4.7)	
	50–59	778(11.5)	591 (12.4)	187 (9.3)	
	60–69	1,313(19.3)	873 (18.3)	440 (21.9)	
	70–79	1,521(22.4)	893 (18.7)	628 (31.2)	
	80–89	838 (12.3)	400 (8.4)	438 (21.8)	
	≥90	84 (1.2)	26 (0.5)	58 (2.9)	
Male		3,913(57.6)	2,783 (58.3)	1,130 (56.2)	0.11
Body mass index at admission, kg/m^2, n (%)	<18.5	976 (14.4)	632 (13.2)	344 (17.1)	<0.001
	18.5–24.9	3,580(52.7)	2,475 (51.8)	1,105 (54.9)	
	25.0–29.9	858 (12.6)	642 (13.4)	216 (10.7)	
	≥30.0	196 (2.9)	161 (3.4)	35 (1.7)	
	Data missing	1,178(17.4)	866 (18.1)	312 (15.5)	
Japan Coma Scale at admission, n (%)	Alert	1,908(28.1)	1,710 (35.8)	198 (9.8)	<0.001
	Dizziness	2,776(40.9)	1,827 (38.3)	949 (47.2)	
	Somnolence	1,089(16.0)	689 (14.4)	400 (19.9)	
	Coma	1,015(15.0)	550 (11.5)	465 (23.1)	
**Comorbidities at admission, n (%)**					
Myocardial infarction		28 (0.4)	12 (0.3)	16 (0.8)	0.001
Congestive heart failure		172 (2.5)	95 (2.0)	77 (3.8)	<0.001
Peripheral vascular diseases		29 (0.4)	20 (0.4)	9 (0.4)	0.87
Cerebral vascular diseases		511 (7.5)	281 (5.9)	230 (11.4)	<0.001
Dementia		257 (3.8)	111 (2.3)	146 (7.3)	<0.001
Chronic pulmonary diseases		188 (2.8)	129 (2.7)	59 (2.9)	0.60
Connective tissue diseases		117 (1.7)	83 (1.7)	34 (1.7)	0.89
Peptic ulcer diseases		334 (4.9)	252 (5.3)	82 (4.1)	0.037

Liver diseases		207 (3.0)	122 (2.6)	85 (4.2)	<0.001
Diabetes mellitus		1,026(15.1)	644 (13.5)	382 (19.0)	<0.001
Chronic renal diseases		130 (1.9)	74 (1.5)	56 (2.8)	<0.001
Malignancy		279 (4.1)	155 (3.2)	124 (6.2)	<0.001
Metastasis		60 (0.9)	32 (0.7)	28 (1.4)	0.004
Weekend admission, n (%)		1,509(22.2)	1,031 (21.6)	478 (23.8)	0.050
Ambulance use, n (%)		3,872(57.0)	2,533 (53.0)	1,339 (66.6)	<0.001
Teaching hospital, n (%)		6,218(91.6)	4,452 (93.2)	1,766 (87.8)	<0.001
Tertiary emergency hospital, n (%)		3,071(45.2)	2,190 (45.9)	881 (43.8)	0.12
Hospital case volume, mean (SD)		24.5 (24.7)	25.2 (25.2)	22.8 (23.3)	<0.001
**Organ support therapies, n (%)**					
ICU/HCU admission		2,220(32.7)	1,438 (30.1)	782 (38.9)	<0.001
Supplemental oxygen		3,340(49.2)	1,982 (41.5)	1,358 (67.5)	<0.001
Mechanical ventilation		807 (11.9)	420 (8.8)	387 (19.2)	<0.001
Catecholamine		529 (7.8)	198 (4.1)	331 (16.5)	<0.001
Renal replacement therapy		113 (1.7)	56 (1.2)	57 (2.8)	<0.001
Blood transfusion		304 (4.5)	125 (2.6)	179 (8.9)	<0.001
**Diagnostic Tests**					
brain MRI		6,340(93.4)	4,413 (92.4)	1,927 (95.8)	<0.001
CSF examination		6,094(89.8)	4,281 (89.6)	1,813 (90.1)	0.56
**Outcomes**					
In-hospital mortality (%)		410 (6.0)	0 (0.0)	-	-
Japan Coma Scale at discharge, n (%)	Alert	4,776(70.4)	4,776 (100.0)	0 (0.0)	-
	Dizziness	1,374(20.2)	0 (0.0)	1,374 (68.3)	
	Somnolence	149 (2.2)	0 (0.0)	149 (7.4)	
	Coma	78 (1.1)	0 (0.0)	78 (3.9)	
	Death	1 (0.0)	0 (0.0)	1 (0.0)	
Disturbance of consciousness at discharge, n (%)		2,012(29.6)	-	2,012 (100.0)	-
Functional Status at discharge (Barthel Index <90), n (%)		2,705(45.5)	1,209 (29.3)	1,496 (81.9)	-
Total hospitalization cost, thousand Japanese yen, median (IQR)		1173.3 (659.3–1975.6)	1008.9 (606.5–1700.8)	1619.0 (924.5–2538.1)	<0.001
Length of hospital stay, days, median (IQR)		25.0 (13.0–45.0)	21.0 (12.0–38.0)	34.5 (17.0–57.0)	<0.001
Transferred to institutional care, n(%)		193 (2.8)	102 (2.1)	91 (4.5)	<0.001

HCU, high care unit; ICU, intensive care unit; IQR, interquartile range; MRI, magnetic resonance imaging.
**P*-values were not calculated for outcomes that define the comparison groups (eg, in-hospital mortality, JCS, and Barthel Index at discharge).
Composite outcome was defined as in-hospital death or a Japan Coma Scale (JCS) score ≥1 at discharge. Patient demographics, clinical factors, and outcomes are compared between patients with and without the composite outcome.

eTable 5. Baseline characteristics of patients stratified by functional outcome (Barthel Index ≤90 at discharge)

**Variables**		Total	BI>90	BI≤90	*P*-value
		N=5,951	N=3,246	N=2,705	
Patients Characteristics					
Age, years, median (IQR)		65.0 (45.0–76.0)	55.0 (36.0–68.0)	73.0 (62.0–81.0)	<0.001
Age category, years, n (%)	0–9	126 (2.1)	28 (0.9)	98 (3.6)	<0.001
	10–19	232 (3.9)	205 (6.3)	27 (1.0)	
	20–29	434 (7.3)	370 (11.4)	64 (2.4)	
	30–39	474 (8.0)	394 (12.1)	80 (3.0)	
	40–49	532 (8.9)	420 (12.9)	112 (4.1)	
	50–59	729 (12.3)	503 (15.5)	226 (8.4)	
	60–69	1,202 (20.2)	683 (21.0)	519 (19.2)	
	70–79	1,387 (23.3)	518 (16.0)	869 (32.1)	
	80–89	755 (12.7)	123 (3.8)	632 (23.4)	
	>90	80 (1.3)	2 (0.1)	78 (2.9)	
Male		3,469 (58.3)	2,000 (61.6)	1,469 (54.3)	<0.001
Body mass index at admission, kg/m^2 , n (%)	<18.5	853 (14.3)	386 (11.9)	467 (17.3)	<0.001
	18.5–24.9	3,312 (55.7)	1,904 (58.7)	1,408 (52.1)	
	25.0–29.9	804 (13.5)	479 (14.8)	325 (12.0)	
	≥30.0	179 (3.0)	123 (3.8)	56 (2.1)	
	Data missing	803 (13.5)	354 (10.9)	449 (16.6)	
Japan Coma Scale at admission, n (%)	Alert	1,618 (27.2)	1,115 (34.3)	503 (18.6)	<0.001
	Dizziness	2,498 (42.0)	1,392 (42.9)	1,106 (40.9)	
	Somnolence	953 (16.0)	441 (13.6)	512 (18.9)	
	Coma	882 (14.8)	298 (9.2)	584 (21.6)	
**Comorbidities at admission, n (%)**					
Myocardial infarction		25 (0.4)	6 (0.2)	19 (0.7)	0.002
Congestive heart failure		161 (2.7)	51 (1.6)	110 (4.1)	<0.001
Peripheral vascular diseases		29 (0.5)	9 (0.3)	20 (0.7)	0.011
Cerebral vascular diseases		461 (7.7)	169 (5.2)	292 (10.8)	<0.001
Dementia		226 (3.8)	50 (1.5)	176 (6.5)	<0.001
Chronic pulmonary diseases		166 (2.8)	73 (2.2)	93 (3.4)	0.006
Connective tissue diseases		106 (1.8)	52 (1.6)	54 (2.0)	0.25
Peptic ulcer diseases		296 (5.0)	167 (5.1)	129 (4.8)	0.51
Liver diseases		192 (3.2)	86 (2.6)	106 (3.9)	0.006
Diabetes mellitus		948 (15.9)	424 (13.1)	524 (19.4)	<0.001

Chronic renal diseases		119 (2.0)	45 (1.4)	74 (2.7)	<0.001
Malignancy		262 (4.4)	100 (3.1)	162 (6.0)	<0.001
Metastasis		56 (0.9)	21 (0.6)	35 (1.3)	0.010
Weekend admission, n (%)		1,307 (22.0)	705 (21.7)	602 (22.3)	0.62
Ambulance use, n (%)		3,397 (57.1)	1,632 (50.3)	1,765 (65.2)	<0.001
Teaching hospital, n (%)		5,417 (91.0)	2,994 (92.2)	2,423 (89.6)	<0.001
Tertiary emergency hospital, n (%)		2,662 (44.7)	1,504 (46.3)	1,158 (42.8)	0.006
Hospital case volume, mean (SD)		24.5 (24.7)	25.6 (25.1)	23.1 (24.1)	<0.001
**Organ support therapies, n (%)**					
ICU/HCU admission		1,983 (33.3)	976 (30.1)	1,007 (37.2)	<0.001
Supplemental oxygen		2,922 (49.1)	1,146 (35.3)	1,776 (65.7)	<0.001
Mechanical ventilation		690 (11.6)	208 (6.4)	482 (17.8)	<0.001
Catecholamine		478 (8.0)	89 (2.7)	389 (14.4)	<0.001
Renal replacement therapy		103 (1.7)	29 (0.9)	74 (2.7)	<0.001
Blood transfusion		265 (4.5)	47 (1.4)	218 (8.1)	<0.001
**Diagnostic Tests**					
brain MRI		5,585 (93.8)	3,012 (92.8)	2,573 (95.1)	<0.001
CSF examination		5,367 (90.2)	2,951 (90.9)	2,416 (89.3)	0.039
**Outcomes**					
In-hospital mortality (%)		410 (6.9)	-	410 (15.2)	-
Japan Coma Scale at discharge, n (%)	Alert	4,124 (69.3)	2,915 (89.8)	1,209 (44.7)	-
	Dizziness	1,218 (20.5)	327 (10.1)	891 (32.9)	
	Somnolence	130 (2.2)	2 (0.1)	128 (4.7)	
	Coma	68 (1.1)	1 (0.0)	67 (2.5)	
	Death	1 (0.0)	1 (0.0)	0 (0.0)	
Composite outcome, n (%)		1,827 (30.7)	331 (10.2)	1,496 (55.3)	-
Functional Status at discharge (Barthel Index <90), n (%)		2,705 (45.5)	-	2,705 (100.0)	-
Total hospitalization cost, thousand Japanese yen, median (IQR)		1184.2 (684.7–1978.1)	933.6 (597.1–1488.2)	1609.5 (935.3–2592.4)	<0.001
Length of hospital stay, days, median (IQR)		25.0 (14.0–45.0)	20.0 (12.0–33.0)	36.0 (18.0–58.0)	<0.001
Transferred to institutional care, n(%)		161 (2.7)	16 (0.5)	145 (5.4)	<0.001

HCU, high care unit; ICU, intensive care unit; IQR, interquartile range; MRI, magnetic resonance imaging.

**P*-values were not calculated for outcomes that define the comparison groups (eg, in-hospital mortality, Japan Coma Scale, and Barthel Index at discharge).

The table compares patient characteristics and in-hospital management between those with preserved function (Barthel Index >90) and impaired function (≤90) at discharge.

eTable 6. Multivariable logistic regression analysis of predictors for the composite outcome (death or impaired consciousness at discharge)

**Variables**	**Odds ratio (95% CIs)**	***P*-value**
Age 0–9	0.20 (0.12–0.33)	<0.001
Age 10–19	0.34 (0.22–0.52)	<0.001
Age 20–29	0.47 (0.34–0.65)	<0.001
Age 30–39	0.71 (0.53–0.96)	0.025
Age 40–49	0.71 (0.55–0.93)	0.012
Age 50–59 (Ref)		
Age 60–69	1.54 (1.25–1.90)	<0.001
Age 70–79	1.96 (1.58–2.42)	<0.001
Age 80–89	2.96 (2.35–3.72)	<0.001
Age ≥90	4.56 (2.61–7.95)	<0.001
Male	0.98 (0.88–1.10)	0.775
BMI <18.5	1.22 (1.04–1.45)	0.017
BMI 18.5–24.9 (Ref)		
BMI 25.0–29.9	0.75 (0.63–0.89)	0.001
BMI ≥30.0	0.60 (0.41–0.87)	0.008
BMI Missing	1.16 (0.95–1.42)	0.134
JCS: Alert (Ref)		
JCS: Dizziness	3.29 (2.77–3.92)	<0.001
JCS: Somnolence	3.54 (2.87–4.38)	<0.001
JCS: Coma	5.51 (4.45–6.83)	<0.001
Myocardial infarction	1.99 (0.91–4.35)	0.086
Congestive heart failure	1.18 (0.83–1.68)	0.366
Peripheral vascular diseases	0.66 (0.31–1.41)	0.280
Cerebral vascular diseases	1.17 (0.96–1.43)	0.113
Dementia	1.69 (1.29–2.19)	<0.001
Chronic pulmonary diseases	0.82 (0.58–1.15)	0.242
Connective tissue diseases	0.73 (0.49–1.10)	0.136
Peptic ulcer diseases	0.82 (0.62–1.07)	0.147
Liver diseases	1.38 (1.01–1.88)	0.043
Diabetes mellitus	1.02 (0.89–1.18)	0.738
Chronic renal diseases	1.38 (0.92–2.06)	0.125
Malignancy	1.46 (1.13–1.88)	0.004
Metastasis	2.02 (1.17–3.48)	0.011
Weekend admission	1.05 (0.93–1.19)	0.410
Ambulance use	1.06 (0.93–1.22)	0.363
Teaching hospital	0.53 (0.40–0.71)	<0.001
Tertiary emergency hospital	1.00 (0.84–1.20)	0.952
Hospital case volume	1.00 (0.99–1.00)	0.685

BMI, body mass index; CI, confidence interval; JCS, Japan Coma Scale.
Adjusted odds ratios and 95% confidence intervals were calculated using generalized estimating equations (GEE), accounting for hospital clustering.

eTable 7. Multivariable logistic regression analysis of predictors for poor functional outcome (Barthel Index ≤90 at discharge)

Variables	Odds ratio (95% CIs)	*P*-value
Age 0–9	8.80 (4.91–15.79)	<0.001
Age 10–19	0.33 (0.22–0.49)	<0.001
Age 20–29	0.45 (0.33–0.61)	<0.001
Age 30–39	0.54 (0.41–0.72)	<0.001
Age 40–49	0.65 (0.49–0.86)	0.003
Age 50–59 (Ref)		
Age 60–69	1.61 (1.31–1.97)	<0.001
Age 70–79	3.29 (2.71–4.00)	<0.001
Age 80–89	9.24 (7.13–11.98)	<0.001
Age ≥90	57.93 (14.61–229.72)	<0.001
Male	0.83 (0.74–0.94)	0.003
BMI <18.5	1.48 (1.22–1.79)	<0.001
BMI 18.5–24.9 (Ref)		
BMI 25.0–29.9	0.89 (0.73–1.08)	0.245
BMI ≥30.0	0.90 (0.63–1.29)	0.580
BMI Missing	1.26 (1.03–1.54)	0.024
JCS: Alert (Ref)		
JCS: Dizziness	1.41 (1.20–1.65)	<0.001
JCS: Somnolence	1.89 (1.56–2.31)	<0.001
JCS: Coma	3.05 (2.47–3.77)	<0.001
Myocardial infarction	2.06 (0.71–5.97)	0.181
Congestive heart failure	1.22 (0.82–1.81)	0.321
Peripheral vascular diseases	1.10 (0.46–2.65)	0.827
Cerebral vascular diseases	1.25 (0.99–1.56)	0.055
Dementia	1.71 (1.23–2.36)	0.001
Chronic pulmonary diseases	1.03 (0.73–1.45)	0.878
Connective tissue diseases	1.08 (0.69–1.72)	0.732
Peptic ulcer diseases	1.09 (0.84–1.42)	0.496
Liver diseases	1.53 (1.13–2.06)	0.006
Diabetes mellitus	1.15 (0.97–1.36)	0.105
Chronic renal diseases	1.44 (0.91–2.30)	0.124
Malignancy	1.31 (0.98–1.75)	0.065
Metastasis	2.00 (1.11–3.60)	0.020
Weekend admission	0.94 (0.81–1.08)	0.361
Ambulance use	1.32 (1.16–1.51)	<0.001
Teaching hospital	0.72 (0.57–0.92)	0.008
Tertiary emergency hospital	1.02 (0.87–1.20)	0.778
Hospital case volume	1.00 (0.99–1.00)	0.282

BMI, body mass index; CI, confidence interval; JCS, Japan Coma Scale.

The table presents independent predictors of functional disability at discharge based on GEE logistic regression modeling.

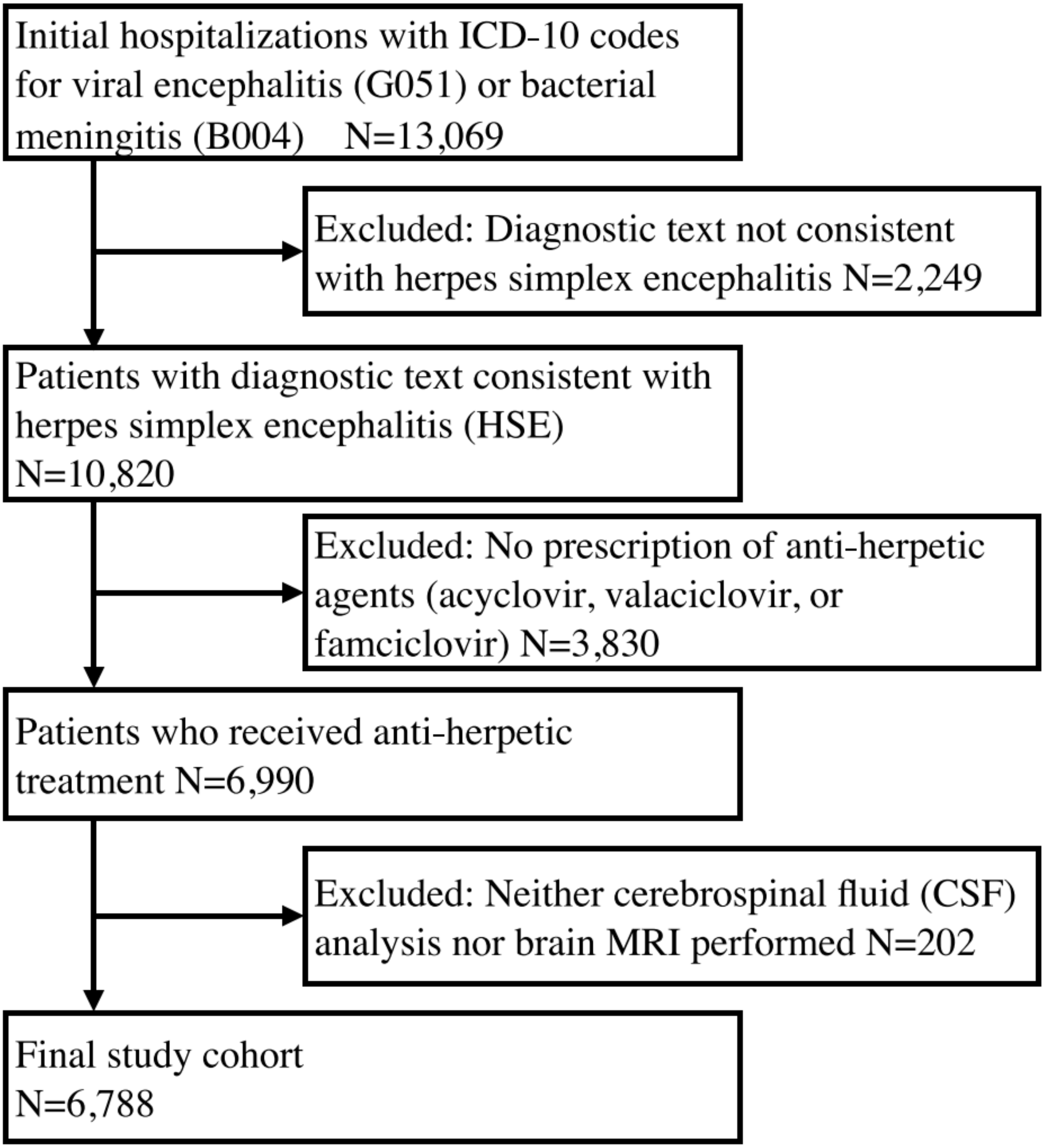

eFigure 1. Flow diagram of patient selection for the study cohort. Patients hospitalized with viral encephalitis or bacterial meningitis were screened using ICD-10 codes and diagnostic text. We excluded patients whose diagnostic text was not consistent with herpes simplex encephalitis (HSE), those who did not receive anti-herpetic treatment, and those who underwent neither cerebrospinal fluid (CSF) examination nor brain magnetic resonance imaging (MRI). The final study cohort included 6,788 patients.

HSE, herpes simplex encephalitis; CSF, cerebrospinal fluid; MRI, magnetic resonance imaging.

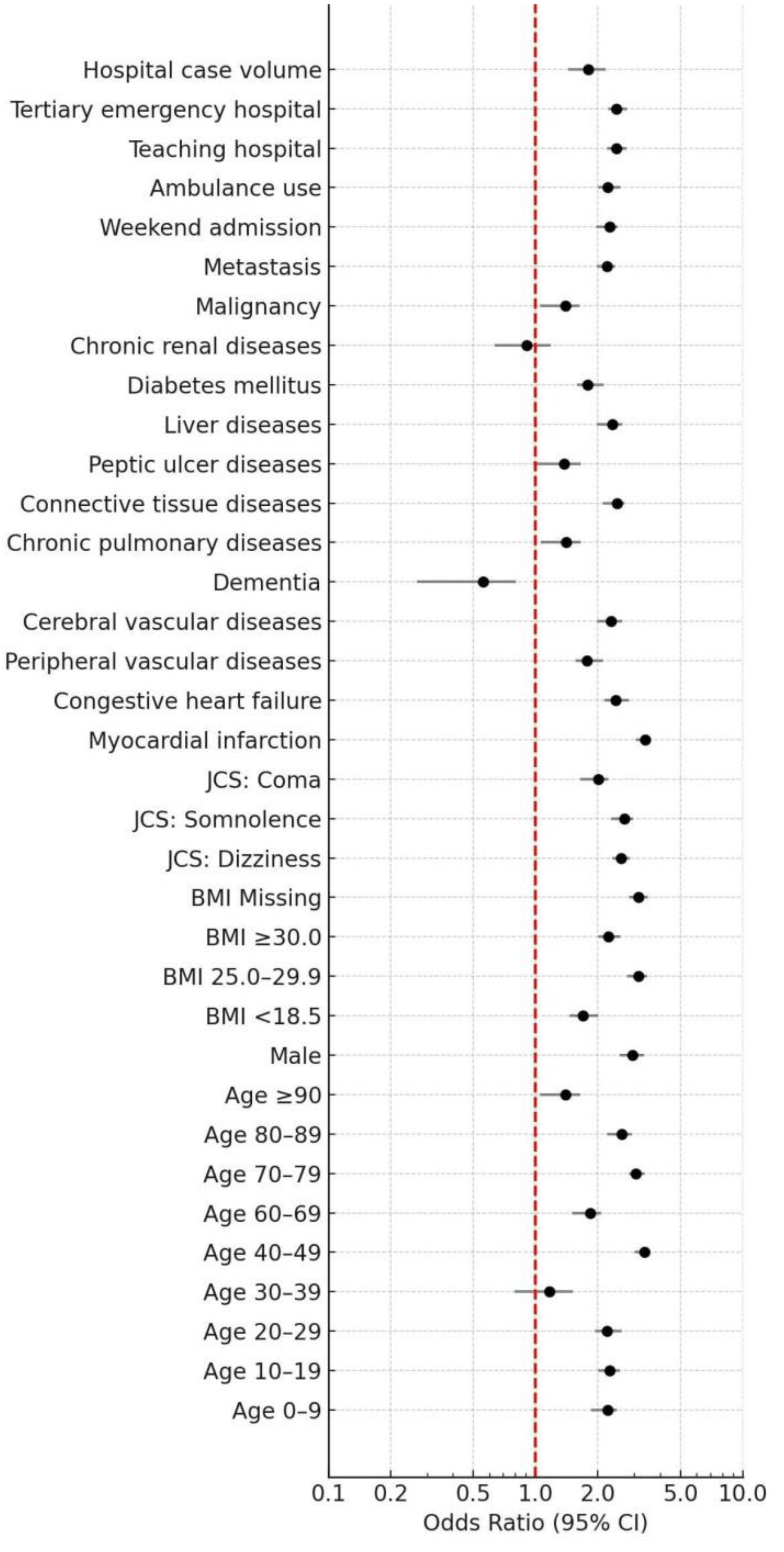

eFigure 2. Forest plot of adjusted odds ratios for the composite outcome in patients with herpes simplex encephalitis. Dots indicate adjusted odds ratios and horizontal lines indicate 95% confidence intervals. The composite outcome was defined as in-hospital death or impaired consciousness at discharge (Japan Coma Scale [JCS] ≥1). Estimates were obtained from generalized estimating equation logistic regression models accounting for within-hospital clustering. Hospital-level variables were included as fixed covariates to adjust for institutional characteristics.

BMI, body mass index; CI, confidence interval; JCS, Japan Coma Scale.

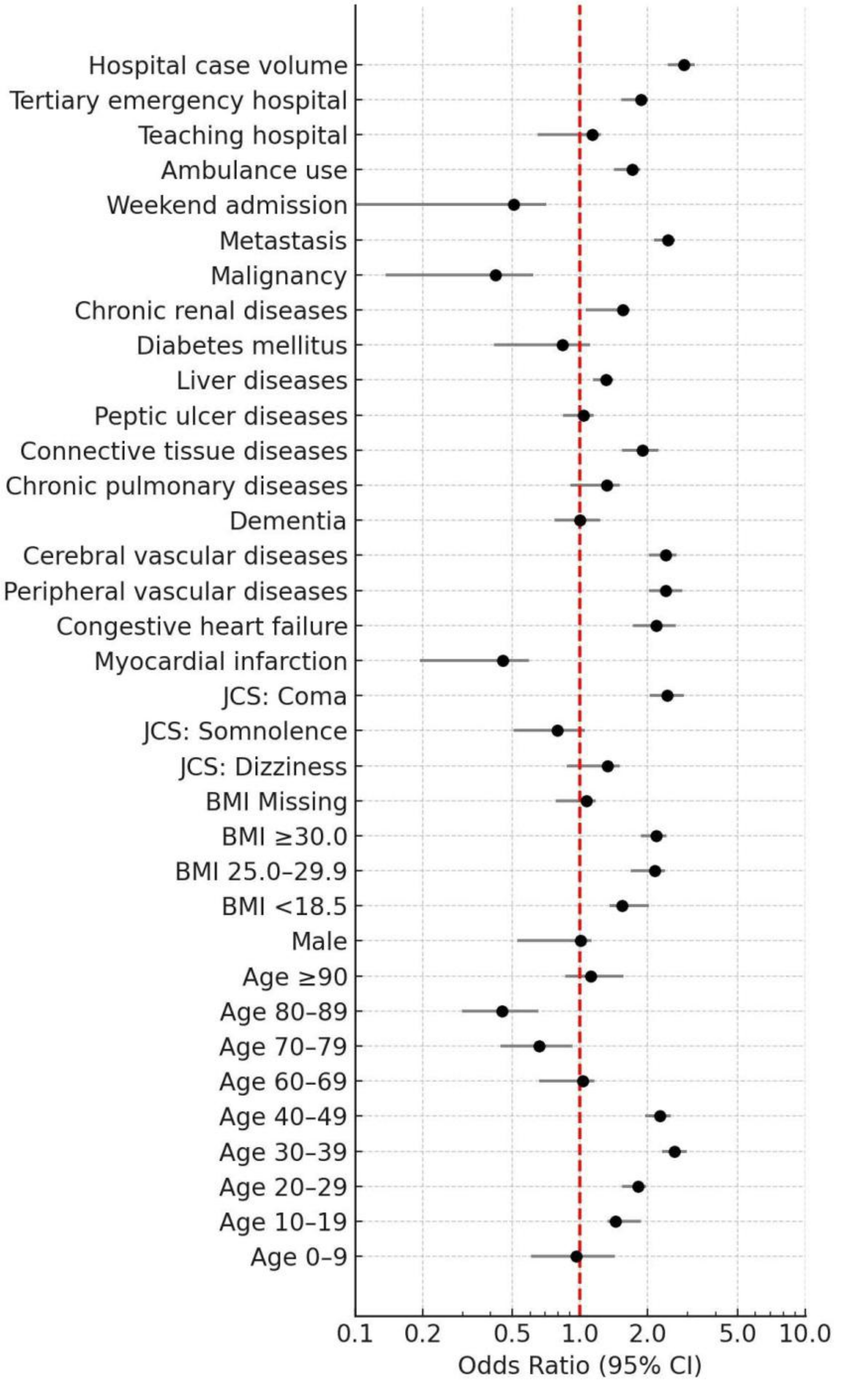

eFigure 3. Forest plot of adjusted odds ratios for poor functional outcome at discharge in patients with herpes simplex encephalitis. Dots indicate adjusted odds ratios and horizontal lines indicate 95% confidence intervals. Poor functional outcome was defined as a Barthel Index ≤90 at discharge. Estimates were obtained from generalized estimating equation logistic regression models accounting for within-hospital clustering. Hospital-level variables were included as fixed covariates to adjust for institutional characteristics.

BMI, body mass index; CI, confidence interval; JCS, Japan Coma Scale.
